# Challenging access during flow diversion treatment of a giant cavernous ICA aneurysm

**DOI:** 10.3171/2022.7.FOCVID2258

**Published:** 2022-10-01

**Authors:** Visish M. Srinivasan, Joelle N. Hartke, Joshua S. Catapano, Ethan A. Winkler, Ashutosh P. Jadhav, Felipe C. Albuquerque, Andrew F. Ducruet

**Affiliations:** Department of Neurosurgery, Barrow Neurological Institute, St. Joseph’s Hospital and Medical Center, Phoenix, Arizona

**Keywords:** aneurysm, microwire, Pipeline

## Abstract

A man in his 60s presented with severe ophthalmoparesis and loss of visual acuity in his right eye. He was found to have a giant aneurysm of the cavernous internal carotid artery (ICA). Treatment with a flow diverter was recommended. The aneurysm caused matricidal outflow restriction of the ICA. Microwire and microcatheter access through the aneurysm was challenging, requiring multiple wires, stentriever reduction, and more. Eventually, a construct of 3 Pipeline embolization devices was created across the aneurysm. Troubleshooting access across giant aneurysms is an important part of treatment. Informed consent was obtained for the procedure and for publication.

The video can be found here: https://stream.cadmore.media/r10.3171/2022.7.FOCVID2258

## Transcript

A man in his 60s presented with 3 to 4 weeks of headaches centered around his right eye, with loss of vision in that eye. His neurological exam, shown here, was significant for severely restricted extraocular movements in his right eye, with loss of acuity as well.

### 0:42 Preoperative Imaging.

Axial noncontrast CT head shows the aneurysm has eroded into the orbit and is exposed to the sphenoid sinus as well.^1^ The giant aneurysm encompasses the cavernous segment of the ICA and even compresses the arterial inflow; thus, we call this a matricidal aneurysm.^2^

### 0:58 Angiography.

Diagnostic angiogram demonstrates the swirling of the contrast around the aneurysm, with the obscuration of the inflow and outflow. Working angle angiogram now shows the inflow and outflow more clearly. It is important to create a barrel view of the outflow to facilitate distal catheterization. There was difficulty on the initial 3D rotational angiography in adequately visualizing the inflow and the outflow due to the altered transit time of the contrast within the aneurysm.

### 1:27 Surgical Strategy.

Here are the key steps to this case. We used transfemoral access and then performed a 3D rotational angiogram with an increased injection delay to capture the contrast as it exited the aneurysm. This facilitated creation of the barrel view. The catheter system was positioned near the aneurysm inflow in a stable position; then we attempted to cross. Initially, we start with the Synchro microwire and attempted to reduce the system prior to device deployment. We switched to the Aristotle microwire and then tried reduction again.^3^ Ultimately, we deployed the first Pipeline device, then reduced the system while deploying the second device. Three devices were overlapped to span the aneurysm.

### 2:05 Synchro Microwire Access.

Our interventional screen shows the PA plane across the top and lateral across the bottom, with fluoroscopy views on the left and roadmap on the right. This is the Synchro 0.014 wire, with 2 curves in the same direction to help access the outflow. We carefully seek the outflow, guided by careful setup of the barrel view working angles. After wire accessing the M1, distal access is much easier, with the Phenom 27 microcatheter tracking out the distal M2. Now we attempt to reduce the catheter. Note how the Phenom takes a circuitous course around the anterior pole of the aneurysm. Before inserting the device, we remove the microwire and pull back on the catheter system. However, rather than reducing the intra-aneurysmal redundancy, we lose our distal purchase. This is a rare scenario. Readvancing the wire opacifies the catheter to visualize our progress of reduction maneuver.

### 3:16 Grappling Hook Technique.

Next, we try the stent retriever as a grappling hook to advance the distal access catheter through the aneurysm and straighten our trajectory across it.^4^ This anchor is usually extremely stable. Here, we access the terminus as we take the turn into the M1 with the distal access catheter. As we apply backward traction on the Catalyst to reduce its curve, we lose distal purchase, and only the microcatheter remains through the aneurysmal outflow.

### 3:57 Aristotle Microwire Access.

Now, we change our system, opting for an Aristotle 24 wire, which has similar softness to the Synchro but slightly more body and less step-off with the microcatheter. Distal access was achieved with more ease, and the microcatheter is positioned in the M2 and the Catalyst distal access catheter in the midaneurysm.

### 4:30 Pipeline Deployment.

The first Pipeline is deployed from the proximal M1 into the aneurysm. We are using the largest Pipeline, which is a 5 × 35–mm device. It deploys with a downward trajectory, but with a solid distal anchor. We maintain wire access through the device and track the microcatheter back out the MCA after the first device is deployed.^5^ As we deploy the second device, in order to bring it off the anteroinferior wall, we pull back on the catheter toward the outflow. In this manner, the 2 devices function as our anchor to reduce the system. As the second device is deployed, we nearly bridge the entire aneurysm. A third device is deployed, solidly anchoring the construct proximally. These 3 devices overlap by approximately 1 cm each. The final construct is relatively reduced, even if not in a straight line from inflow to outflow.

### 6:05 Postdeployment Images.

Here are the postdeployment images, showing the telescoping construct, with overlap of 1 cm between each device. Each of the individual colors of the lines represents a single device. These lateral and PA images show stagnation of the contrast within the aneurysm with satisfactory distal flow through the construct. This illustration of the carotid siphon shows the cavernous segment of the ICA, encased within the cavernous sinus, with the potential for compromising outflow in the C5 and C6 segments as it grows.

### 6:37 Advanced Access Maneuvers.

There are several learning points to this case, primarily around the concept of access. Access is critical in treating giant aneurysms. Here, the careful shaping or reshaping of the wire, with a double forward angle hook, facilitated access to the outflow. With the Aristotle wire, the controlled torque allows us to seek the outflow, and then track the 0.027 microcatheter over it without step-off. Alternatively, one can try the “around the world” technique, where encircling the aneurysm leads to finding the outflow. However, this is not preferred, as it may be hard to reduce the wire without losing distal access. Once we had the catheter access, we removed the wire to reduce the catheter. While this was not successful in this case, it is important to remove stiffness from the wire in order to allow the redundancy of the catheter to be removed. We also attempted using the grappling hook technique, which can help support larger distal access catheters through the outflow of the aneurysm. Alternative techniques, such as balloon anchoring or deflection, have been described but were not used in our case.

### 7:39 Stent Retriever Applications.

Stent retrievers are a key tool in our endovascular toolkit. While primarily used for mechanical thrombectomy, they have numerous other applications, as shown here, including catheter reduction and the grappling hook technique.

### 7:52 Outcome.

The patient had no new neurological issues postoperatively and was discharged home. He is pending follow-up angiogram.

### 7:58 Conclusions.

In conclusion, catheterizing outflow arteries in giant aneurysms can be challenging. Varying microwires, positioning of catheter systems, or using varied reduction techniques is important to achieve a stable flow diversion construct. Building a flow diversion construct from distal to proximal is helpful when the outflow is challenging to access. Thank you.

## Acknowledgments

We thank the staff of Neuroscience Publications at Barrow Neurological Institute for assistance with manuscript and video preparation.

## Disclosures

Dr. Ducruet: consultant for Stryker, Medtronic, Penumbra, Cerenovus, Koswire, and Oculus; and ownership interest in Aneuvas Technologies.

## Author Contributions

Primary surgeon: Ducruet. Assistant surgeon: Srinivasan. Editing and drafting the video and abstract: Srinivasan, Hartke. Critically revising the work: Ducruet, Srinivasan, Winkler, Jadhav, Albuquerque. Reviewed submitted version of the work: all authors. Approved the final version of the work on behalf of all authors: Ducruet. Supervision: Ducruet, Jadhav, Albuquerque.

## Supplemental Information

### Patient Informed Consent

The necessary patient informed consent was obtained in this study.
